# Properties of the exotic metastable ST12 germanium allotrope

**DOI:** 10.1038/ncomms13909

**Published:** 2017-01-03

**Authors:** Zhisheng Zhao, Haidong Zhang, Duck Young Kim, Wentao Hu, Emma S. Bullock, Timothy A. Strobel

**Affiliations:** 1 Geophysical Laboratory, Carnegie Institution of Washington, Washington, District of Columbia 20015, USA; 2 State Key Laboratory of Metastable Materials Science and Technology, Yanshan University, Qinhuangdao 066004, China; 3 Center for High Pressure Science and Technology Advanced Research, 1690 Cailun Road, Building 6, Pudong, Shanghai 201203, China

## Abstract

The optical and electronic properties of semiconducting materials are of great importance to a vast range of contemporary technologies. Diamond-cubic germanium is a well-known semiconductor, although other ‘exotic' forms may possess distinct properties. In particular, there is currently no consensus for the band gap and electronic structure of ST12-Ge (*tP*12, *P*4_3_2_1_2) due to experimental limitations in sample preparation and varying theoretical predictions. Here we report clear experimental and theoretical evidence for the intrinsic properties of ST12-Ge, including the first optical measurements on bulk samples. Phase-pure bulk samples of ST12-Ge were synthesized, and the structure and purity were verified using powder X-ray diffraction, transmission electron microscopy, Raman and wavelength/energy dispersive X-ray spectroscopy. Optical measurements indicate that ST12-Ge is a semiconductor with an indirect band gap of 0.59 eV and a direct optical transition at 0.74 eV, which is in good agreement with electrical transport measurements and our first-principles calculations.

## Abstract (web summary)

There has been disagreement on the properties of the ST12 phase of germanium due to the purity and size scale of samples synthesized so far. Here authors demonstrate a method for making pure, bulk samples and measure properties in agreement with computational predictions.

As a widely used semiconductor material^1^, germanium (Ge) crystallizes in the diamond-cubic structure (DC-Ge) with an indirect band gap of 0.66 eV at standard conditions^2^. Under pressure, however, DC-Ge undergoes a series of structural transitions, which have been the subject of a large number of theoretical and experimental studies^3^. Ge first transforms to the metallic β-Sn (*I*4_1_*/amd*) structure at ∼10-11 GPa (ref. 4), to an intermediate orthorhombic *Imma* phase at ∼75 GPa (ref. 5) and then to the simple hexagonal (sh) structure between 80 and 90 GPa (refs 6, 7, 8). At ∼100 GPa, Ge assumes a *Cmca* phase (an orthorhombic structure with 16 atoms per unit cell)^7^ and finally transforms to the hcp structure at 160–180 GPa (ref. 7). Decompressed metallic Ge does not follow the reverse structural sequence observed during compression; metastable back-transformations lead to new allotropes, the formation of which depends on the speed of pressure release. Fast decompression from the β-Sn-Ge phase results in the formation of BC8 (*Ia*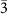) Ge^9^,^10^,^11^,^12^, which gradually changes to the hexagonal diamond (*P*6_3_*/mmc*) structure at ambient conditions^9^,^10^,^13^,^14^. Slow decompression leads to the metastable tetragonal ST12 structure (also called Ge-III)^4^,^15^,^16^,^17^, which contains 12 atoms per unit cell in the space group *P*4_3_2_1_2 (*D*^8^_4_) (refs 16, 18). It was also reported that decompression from pressure-induced metallization of amorphous Ge results in the formation of both BC8 (refs 13, 19, 20) and ST12 (ref. 21) phases. The R8 (*R*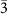) phase has also been observed during decompression^14^,^15^,^20^ and hydrostaticity has been suggested as an important parameter for phases selection^22^. At ambient pressure, chemical methods have been used to produce Ge in the form of clathrates^23^, 4H and so-called allo-Ge^24^,^25^,^26^. As members of elemental group IV, similar structures are often observed for both Ge and Si, for example, DC, BC8/R8 and ST12. Nevertheless, differences are observed during decompression and the tendency for Ge to form ST12 (opposed to BC8/R8 for Si) may be rooted in kinetic origins^27^. In addition, experimental evidence for new Si allotropes formed through ultrafast laser-induced microexplosions also suggest promising pathways to access new forms of Ge^28^.

Among all the Ge allotropes that are metastable at ambient conditions, ST12-Ge attracts attention for potential electronic or energy applications. Bundy and Kasper^16^ first observed this phase with a packing density about 11% greater than that of the DC-Ge phase in compression and unloading experiment in 1963. It has a distorted tetrahedral arrangement with the atoms located in fivefold, sixfold and sevenfold rings. The complex structure has a range of bond lengths and a distribution of bond angles: the three unique bond distances between the two unique atoms in the unit cell vary between 2.4807(8) and 2.5053(6) Å, whereas bond angles vary between 101.15(5) and 136.25(2) degrees^29^.

Both theoretical and experimental efforts were performed to reveal the properties of ST12-Ge. The reported results, however, vary greatly with regards to the electronic band structure and thermal stability. Using empirical pseudopotentials with a tight-binding model, Joannopoulos and Cohen^17^ calculated that ST12-Ge has a direct band gap of 1.47 eV, which indicates great potential as a solar absorber material. Mujica and Needs^18^ performed *ab initio* calculations within the local-density approximation (LDA) using a plane-wave pseudopotential approach and reported a direct band gap of 0.7 eV; however, this value was regarded as a lower limit as density functional theory (DFT) typically underestimates band gaps^30^. Most recently, Malone and Cohen^31^ also used LDA-based DFT to predict an indirect fundamental band gap of 0.54 eV and a direct non-fundamental gap of 0.56 eV. The difference in the nature of the fundamental gap was attributed to a finer sampling of the Brillouin zone (BZ) and the magnitude of the gap was again regarded as a lower bound given the reason stated above.

Experimentally, although many researchers were able to access this phase through various methods, for example, diamond anvil cell experiments^4^,^21^,^22^,^32^, thermal annealing from amorphous nanophase Ge by the naphthalide-mediated reduction of GeCl_4_ and subsequent treatment with t-BuMgCl^33^, or the cluster-beam evaporation technique^34^, accurate electrical and optical measurements are difficult, primarily due to lack of bulk samples. Thus far, most of the experimental studies have been limited to nano- or micrometre-sized samples. We are aware of only one report on synthesis of single crystal ST12-Ge (40 × 50 × 25 μm^3^) through high-pressure plus high-temperature annealing using a multi-anvil module^29^; however, no optical, Raman or thermal stability properties were investigated. Moreover, due to non-ohmic contacts and sample geometry, the inaccuracy of the absolute electrical resistivity was estimated to be up to 50%. The resulting estimated lower limit of the energy gap of 0.35 eV may also be reduced with respect to the fundamental band gap due to defects and impurities^29^. To our best knowledge, the only other reported experimental study on the band gap of ST12-Ge was through optical absorption measurements on nanocrystals that were deposited by the cluster-beam evaporation technique with a size of 3–4 nm (ref. 35). This study concluded that the band gap for ST12-Ge nanocrystals is about 1.5 eV; however, no optical measurements have been obtained thus far on bulk samples and the intrinsic bulk properties remain uncertain. Notably, the tetragonal Ge nanoparticles produced by the gas evaporation technique^36^ may be more consistent with a new configuration rather than ST12-Ge^37^.

The metastable phase of ST12-Ge transforms into the DC-Ge structure at high temperatures. The previously reported transition temperatures, however, are not consistent. It was reported that the ST12 phase obtained from the high-pressure route transformed after annealing at 473 K for 6 h (refs 16, 38). Nanocrystalline ST12 from thermal annealing of the amorphous nanophase Ge was reported to persist at a temperature up to 773 K (ref. 33) and ST12 nanoparticles from cluster beam evaporation persisted after annealing between 973 and 1073 K (ref. 35).

Here we demonstrate the successful synthesis of bulk (millimeter sized), phase-pure samples of ST12-Ge, which allow for accurate properties characterization. The structure and purity were confirmed through X-ray diffraction (XRD), transmission electron microscopy (TEM), Raman and wavelength/energy dispersive X-ray spectroscopy. The optical and electrical properties were obtained through optical absorption/reflectivity and electrical conductivity measurements, and first-principles calculations with hybrid pseudopotentials and high-resolution sampling of reciprocal space were performed to better elucidate the nature of the electronic band structure.

## Results

### Synthesis

**Figure 1:**
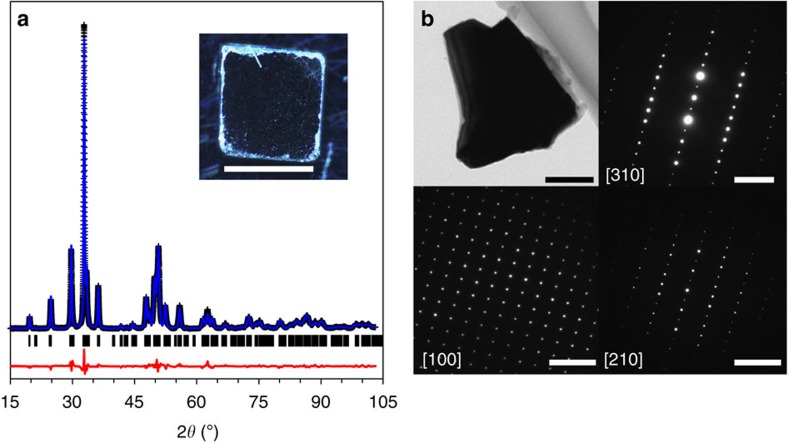
Structure of ST12-Ge. (**a**) Experimental PXRD pattern (Cu K_α_) of ST12-Ge (black points) and Rietveld refinement (blue line), with difference (red line). *wR*_*p*_-background=5.08%, *R*_p_-background=3.84%, *χ*^2^=1.15. The inset shows an image of the recovered ST12-Ge sample polished as a bar (scale bar, 2 mm). (**b**) TEM image (scale bar, 100 nm) of single crystalline grain extracted from the bulk sample and corresponding electron diffraction patterns along different zone axes (scale bars, 5 nm^−1^).

Bulk samples of ST12-Ge were synthesized using the multi-anvil press method^39^. Ge powder (Alfa Aesar, 99.999%) was pressed into a hexagonal boron nitride capsule and loaded within a COMPRES-style 14/8 multi-anvil assembly^40^. Approximately 15 mg pellets of ST12-Ge were obtained after slow decompression from 14 GPa (at room temperature) to atmospheric pressure. The recovered samples consisted of fully dense polycrystalline ingots (∼2 mm in diameter and length), which were readily polished into bars as shown in Fig. 1.

### Structure and purity identification

The samples were first characterized through powder XRD (PXRD). Phase-pure ST12-Ge was verified and no DC-Ge or other phases were detectable using PXRD, as shown in Fig. 1a. Rietveld refinement of the experimental data indicates structural parameters that are in very good agreement with previous results^16^,^41^,^42^. The lattice parameters of the tetragonal unit cell were determined to be *a*=5.93284(6) Å and *c*=6.9797(1) Å, with the two crystallographically distinct atoms (Ge1 and Ge2) located at *4a*: *x*_1_=0.0901(1) and *8b*: *x*_2_=0.17196(9), *y*_2_=0.3724(1), and *z*_2_=0.2566(1). Scanning electron microscopy and TEM images showed typical grain sizes ranging from ∼100 nm to ∼5 μm. Wavelength dispersive and energy dispersive X-ray spectroscopy measurements were performed to investigate the chemical purity after the high-pressure transformation and indicated that the samples were pure Ge (100.06%, *N*=9) within the instrumental detection limits. Selected area electron diffraction measurements on individual grains recovered after mechanical agitation could only be indexed to the ST12 structure (Fig. 1b), providing further support for the phase purity of the recovered samples.

### Raman spectrum

**Figure 2:**
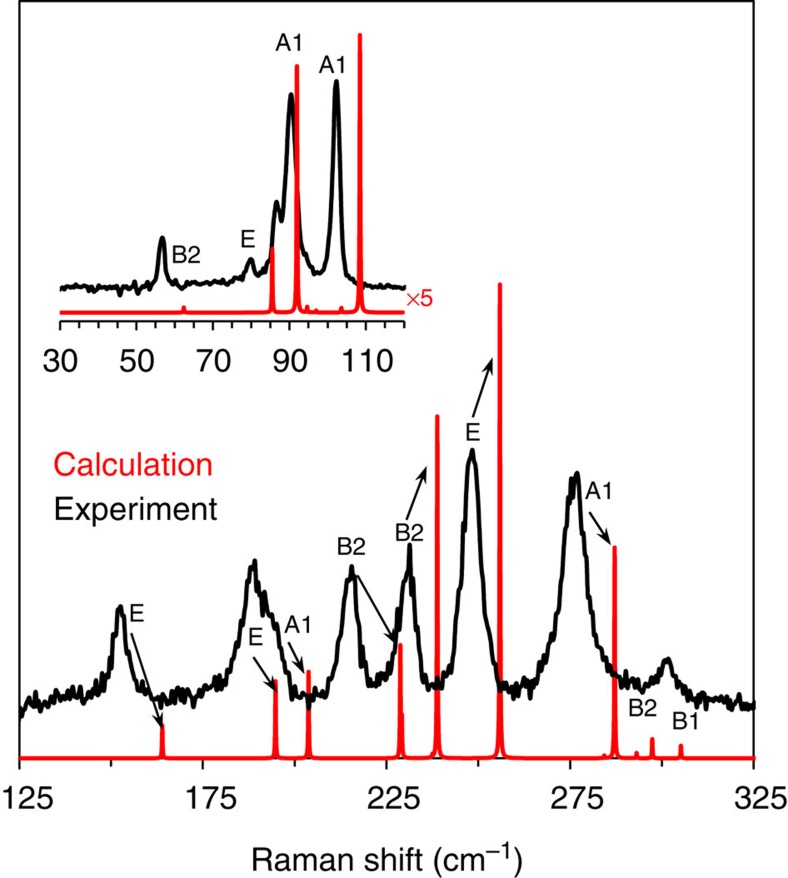
Raman spectrum of ST12-Ge compared with DFT calculations. The calculated peaks are shown as Lorentzian profiles with an arbitrary width.

The ST12-Ge phase has a rich Raman spectrum^32^,^38^,^41^ and is reported here in the low-frequency region at ambient pressure for the first time. Group theoretical analysis of the *P*4_3_2_1_2 structure reveal 4A_1_+5B_1_+4B_2_+8E Raman active optical phonon modes. At least 12 of these characteristic peaks are clearly observed for the recovered sample at room temperature (Fig. 2) and are in general agreement with previous reports above ∼100 cm^−1^ (refs 38, 41). No peaks corresponding to DC-Ge or any other impurities were detected from the Raman spectroscopy data, further demonstrating phase-pure ST12-Ge samples. The Raman lines are relatively broad, with half-widths near 15 cm^−1^. This linewidth broadness is consistent with other reports and could be an effect related to residual stress^38^ or grain size (a fraction of grains below ∼100 nm were observed in TEM images). To assign specific symmetries to the modes observed, we have performed density functional perturbation theory calculations and have also calculated Raman intensities. There is general agreement between the experimental and calculated spectra and all calculated modes with appreciable intensity are observed experimentally. However, there is a nearly systematic 6 cm^−1^ red shift in the calculated frequencies when compared with experiment. This is probably caused by subtle differences in the calculated lattice parameters and atomic coordinates, which were relaxed using Perdew, Burke, Ernzerhof (PBE)-based generalized gradient approximation for exchange-correlation energy. We note that the phonon frequencies calculated by Malone and Cohen^31^ using LDA are in better quantitative agreement with our experimental data; PBE lattice parameters obtained here show an average deviation of +1.9%, whereas LDA parameters deviate by an average of −1.7%.

### Experimental band gap

**Figure 3:**
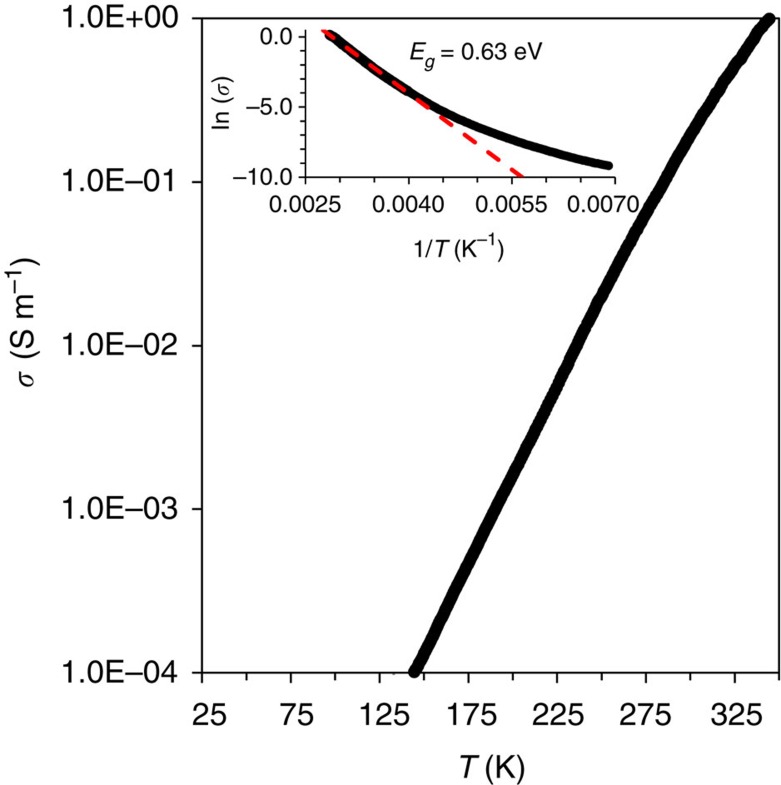
Electrical conductivity of ST12-Ge. Temperature dependence of the electrical conductivity for ST12-Ge. Inset shows the activation energy fit to the high-temperature data.

The temperature-dependent electrical conductivity of a polished sample was measured from 350 K down to 150 K (below this temperature the resistance was too high to measure). The temperature dependence of electrical conductivity for ST12-Ge is shown in Fig. 3. The conductivity increases with temperature over the entire measurement range, indicating clear semiconductor behaviour. The magnitude of the ST12-Ge conductivity at 300 K (0.2 S m^−1^) is comparable to that of DC-Ge. The band gap for an intrinsic semiconductor can be approximated from the thermally activated nature of conduction, 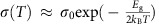, where *σ(T)* is the temperature-dependent electrical conductivity, *σ*_0_ is a constant, *E*_g_ is the band gap, *k*_B_ is Boltzmann's constant and *T* is the temperature. By fitting the conductivity data in the high-temperature region between 350 and 250 K (Fig. 3), the band gap for ST12-Ge was estimated to be 0.63 eV. We assume that this temperature range is representative of intrinsic conduction based on previous observations in pure DC-Ge^43^ and the observed Arrhenius linearity over this temperature range. By varying the fitting across different ranges between 250 and 350 K, the maximum change in the extracted band gap is only 0.01 eV. Departure from this linearity was observed below 250 K, indicating a transition in the nature of conduction and the importance of a different scattering mechanism, but no regime of constant conductivity was observed at the lowest temperatures where measurements were possible.

**Figure 4:**
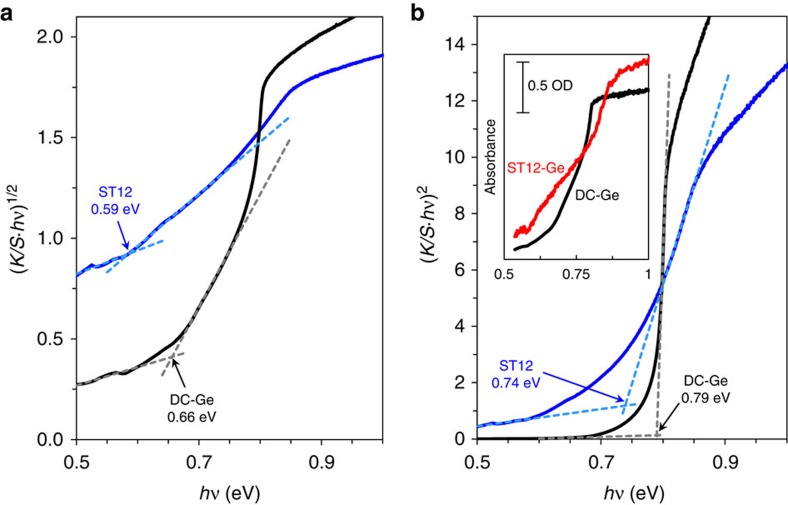
Optical properties of ST12-Ge. Tauc plots of the Kubelka–Munk absorption derived from diffuse reflectance for ST12-Ge and DC-Ge for (**a**) indirect allowed transitions and (**b**) direct allowed transitions. The inset in **b** shows the absorbance derived from transmission measurements using ∼3 wt% ST12- and DC-Ge in KBr.

To further probe the nature of the band gap, optical absorbance and reflectivity measurements of ST12-Ge were performed on powdered samples. We also measured powder samples of the DC-Ge starting material for comparison, which serves as a well-known reference for the measurement system. The general absorbance trends for both ST12-Ge and DC-Ge (as measured by transmission through a ∼3 wt% Ge in KBr pellet) are quite similar: they both display a broad absorbance onset near 0.6 eV (indicative of an indirect gap), followed by a sharper optical transition at higher energy. Tauc plot^44^ analysis of the Kubelka–Munk absorbance 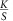, the ratio of absorption and scattering coefficients, derived from diffuse reflectance measurements on pure ST12- and DC-Ge powders are shown in Fig. 4. The indirect and direct gap features are indicated by the plots of 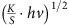 and 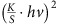 versus *hv*, respectively.

Using this approach, the indirect (fundamental) and direct band gaps of DC-Ge (powder, Alfa Aesar) were determined to be 0.66 and 0.79 eV, respectively, in good agreement with literature values^2^. The Tauc plots for ST12-Ge show a fundamental indirect band gap of 0.59 eV and a direct transition at 0.74 eV. The band gaps derived from reflectivity measurements for both ST12- and DC-Ge were reproduced within 0.02 eV for three independent samples and variation within this bound was obtained from reflectivity and transmission measurements performed on the same sample.

### Theoretical band structure

To gain deeper insights into the electronic band structure, we have performed DFT calculations using the HSE06 hybrid functional and the GW approach to estimate accurate band dispersion. With a uniform grid of 40 × 40 × 40 in *k*-point sampling, we observed that the valence band maximum (VBM) is not located along a high-symmetry line and the conduction band minimum (CBM) is located along the M to Γ direction, which is consistent with the previous calculations of Malone and Cohen^31^. Malone and Cohen^31^ used 60 × 60 × 60 *k*-point sampling grid to find the VBM at (0.316, 0.333, 0.083) and the CBM at (0.333, 0.345, 0). They also reported the indirect bandgap of 0.54 eV and direct bandgap of 0.56 eV using LDA-based DFT. To accurately determine the positions of VBM and CBM, we conducted higher-resolution band structure calculations, sampling only this particular region of the BZ, which gave an equivalent *k*-point sampling of 100 × 100 × 100 points on a uniform grid.

**Figure 5:**
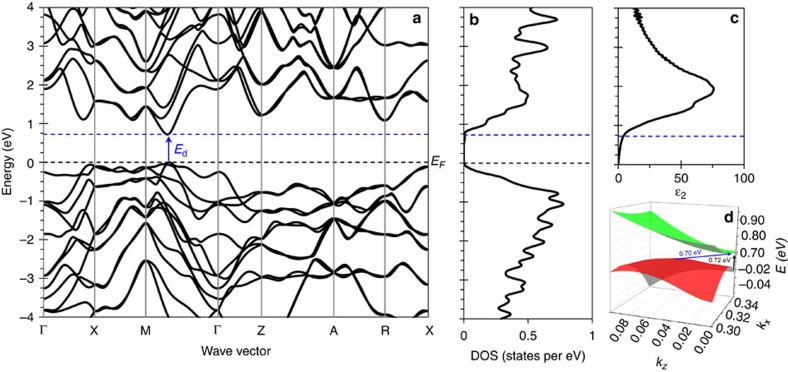
Electronic structure of ST12-Ge. (**a**) Electronic band structure along high-symmetry lines, (**b**) electronic density of states, (**c**) imaginary dielectric function from the Bethe–Salpeter equation (BSE) calculation and (**d**) a schematic diagram of the valence and conduction bands. It is worth noting that the fundamental indirect gap is not along a high-symmetry direction.

From detailed analysis of the band structure (Fig. 5a), we find that the fundamental gap is indirect with a magnitude of 0.70 eV. The VBM is located at (0.340, 0.330, 0.060) and the CBM is located at (0.350, 0.350, 0). The VBM is not located along a high-symmetry direction in the BZ and a very dense grid of *k*-points is needed to accurately find it. This means that the calculated density of states (Fig. 5b) is quite sensitive to the sampling of BZ. The calculations also reveal a direct gap at (0.350, 0.350, 0) with a magnitude of 0.72 eV (HSE06). To predict optical spectra of ST12-Ge, we also calculated the imaginary portion of the dielectric function with respect to frequency (*ɛ*_2_) using the Bethe–Salpeter equation level of approximation as shown in Fig. 5c. The calculated onset of absorption is in good agreement with the position of the non-fundamental direct band gap and slight pre-edge absorption is attributed to excitonic effects and indicates that the transition is dipole allowed. The calculated fundamental indirect gap and non-fundamental direct gap (illustrated in Fig. 5d) are in good agreement with both the result of Malone and Cohen^31^, and the experimental data, with the absolute difference in magnitude varying by ∼0.1 eV, which may be attributed to thermal effects not taken into account in the calculations^45^.

### Thermal stability

**Figure 6:**
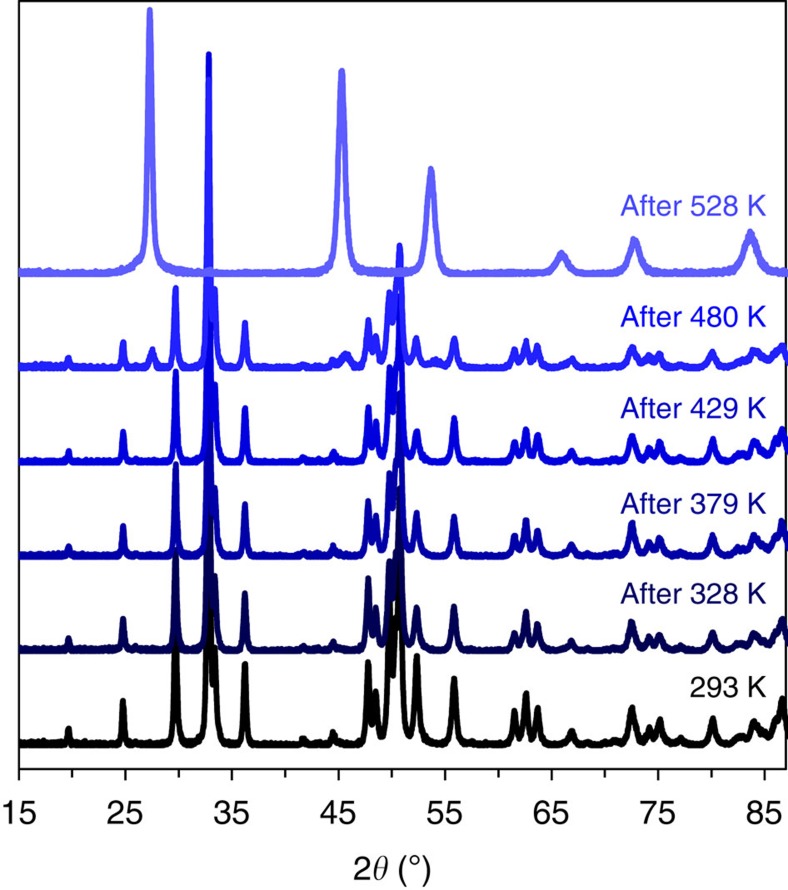
Thermal stability of ST12-Ge. The plot compares the PXRD patterns of ST12-Ge at room temperature before and after heating at various elevated temperatures for 1 h. After heating at 480 K, the XRD shows a mixture of ST12- and DC-Ge phases; after further heating at 528 K, the sample was fully converted to DC-Ge.

Given the wide variation in previous reports, the thermal stability for bulk ST12-Ge was also examined to determine the true stability. Using steps of ∼50 K, ST12-Ge was heated for an hour in open air, then removed from the furnace, cooled down in air and examined by XRD. We chose to heat the samples in air (as opposed to an inert environment) to compare with previous studies. The result shows that after heat treatment at 480 K, the XRD started showing a mixture of DC-Ge and ST12 phases, although the majority was still the ST12 phase. After 528 K, the sample was completely converted to DC-Ge, as shown in Fig. 6.

The previous range of phase transition temperatures for ST12- to DC-Ge range from 473 K to ∼1,073 K (refs 16, 33, 35, 38). It is noted that smaller particles tend to have a greater transition temperature. Kim *et al*.^33^ suggested that this phenomenon is related to the surface energy and surface tension based on the estimation from Bottomley *et al*.^46^ that the surface energy of a 4 nm particle is on the order of 1 J m^−2^ corresponding to an internal pressure of 1 GPa. We attributed the discrepancy to the size of crystal and the related surface energy and surface tension: the nanocrystal tends to have a higher thermal stability temperature than that in the bulk sample. From our results, we thus conclude that bulk samples (with average grains >100 nm) exhibit an intrinsic transition temperature near 480 K.

## Discussion

We successfully synthesized pure bulk ST12-Ge, which provided the basis for definitive characterization and insights for potential future applications. The synthesis method of cold compression plus slow decompression demonstrated a simple pathway for large sample production. Through optical experiments, we found that the ST12 phase exhibits an indirect band gap of 0.59 eV and direct optical transition of 0.74 eV, which is in quantitative agreement with electrical conductivity measurements (0.63 eV) and DFT-based first-principles calculations using the GW approximation for self-energy and hybrid pseudopotentials for exchange correlation energy. Comparing the optical and electronic properties of the DC and ST12 structures of Ge, they are quite similar in terms of indirect and direct transitions with the ST12 structure featuring a slightly smaller band gap. These features probably eliminate the possibility of single-junction solar absorber applications, but the smaller indirect and direct gaps may suggest ST12-Ge to be a better material for infrared detection and imaging compared with the DC phase, and may also indicate potential for both high-frequency and low-voltage electronic applications.

The bulk thermal stability was also examined and ST12-Ge starts to transform to the thermodynamically stable DC-Ge structure near 480 K. This result clarifies existing differences between bulk and nano-scale samples and places strict bounds on the intrinsic properties. In the future, it would be of interest to explore the detailed kinetics of this phase transition.

Overall, the cohesive synergy between experiment and theory paints a clear picture of the intrinsic properties of ST12-Ge, which is in contrast with the commonly-held perception of a fundamental direct band gap near 885 nm. The current study ends a long-standing debate and solves the important problem of the band gap and electronic structure, as well as thermal stability for this exotic phase of Ge.

## Methods

### Sample synthesis and structure

Bulk samples of ST12-Ge were synthesized using a 1,500-ton Kawai-type multi-anvil press. About 15 mg pure Ge powder (Alfa Aesar, 99.999%) was filled inside a cylindrical boron nitride capsule (about 3 mm diameter and 4 mm height), which was at the centre of a cylindrical ZrO_2_ sleeve with MgO spacers on both ends. The Ge powders were separated by a >99% pure pressure-sealed hexagonal BN capsule (with Boric Oxide <0.1%), preventing any impurities being mixed in from the pressurizing process. The assembly was then inserted into a 5% Cr_2_O_3_-doped MgO octahedra with 14 mm edge length, which served as the pressure-transmitting medium. The octahedra centered against eight tungsten carbide anvils with 8 mm truncation edge lengths. The pressure was calibrated through monitoring phase transitions of certain materials: quartz to coesite, CaGeO_3_ garnet to perovskite, coesite to stishovite and olivine to wadsleyite from quenched experiments^40^. The sample was compressed to 14 GPa at a rate of 0.7 GPa h^−1^ at room temperature. The pressure was held still for 12 h, then decompressed to atmospheric pressure at the same rate of −0.7 GPa h^−1^.

### X-ray diffraction

The recovered sample was characterized through XRD (Bruker D8 Discover) equipped with a microfocus source (Cu K_α_) and Vantec500 area detector. Rietveld refinement was performed using GSAS^47^ with EXPGUI^48^.

### Raman spectroscopy

Raman spectra were obtained using a micro-optical system using the back-scattering geometry at room temperature. The Raman system used a 532 nm green laser as the excitation source and was equipped with liquid-nitrogen-cooled charge-coupled device detector. The scattered light was processed by Princeton Instruments Acton SP 2750 spectrograph. The laser power was maintained at approximately 1 mW on the sample with a spot size of about 15 μm^2^. An 1800, grooves/mm grating was used to achieve ∼1 cm^−1^ resolution.

### Optical reflectivity and absorption

The band gap of ST12-Ge was obtained through optical diffuse reflectance and absorbance measurements. The samples were first ground into powders, which were either spread onto a black sample holder with an approximate diameter of 10 mm for reflection or pressed into ∼3 wt% KBr pellets for transmission. The samples were placed at one end of a 150 mm integrating sphere with an InGaAs detector (Perkin Elmer, Lambda 950). Band gaps were obtained from Tauc plots of Kubelka–Munk absorption. To ensure the validity of the measurement procedure, samples of the starting DC-Ge powder were prepared the same way and measured band gaps were obtained in good agreement with accepted values.

### Electrical measurements

The temperature-dependent four-probe electrical conductivity for ST12-Ge was measured through a Physical Property Measurement System from Quantum Design. Platinum wires were attached to the sample surface (∼2 × 2 mm^2^) using Leitsilber conductive silver cement (Ted Pella, silver content 45%, sheet resistance: 0.02–0.04 Ω □^−1^).

### Thermal stability

The ST12-Ge samples were heated under air in steps of 50 K in a furnace for a duration of 1 h per step. After each step, the sample was then taken out of the oven, cooled down in air and analysed by PXRD.

### Transmission electron microscopy

The bright-field TEM image and selected area electron diffraction patterns of the recovered sample were characterized by a Titan ETEM G2 at Yanshan University with an accelerating voltage of 300 kV. To prepare a TEM sample, the recovered sample was crushed and ground in the agate mortar. The crushed small particles were dispersed in an ethanol solution using anultrasonic bath and drop-casted on a carbon coated copper grid, and then dried for TEM analysis.

### Electron microprobe

The samples were analysed using a JEOL 8530F electron probe, equipped with a Thermo Scientific energy-dispersive spectroscopy (EDS) detector operating the Noran System 7 software. The operating conditions were 15 kV and 10 nA for both EDS and wavelength-dispersive spectroscopy analyses. The count times for EDS analyses were 60 s live time and the spectrum was examined for any peaks other than Ge. For wavelength-dispersive spectroscopy analyses, the count time was 20 s on-peak and 10 s for each background measurement, with pure Ge used as the standard.

### First-principles calculations

Electronic structure calculations and Raman intensity calculations were performed using DFT^49^,^50^ as implemented in the VASP software package^51^ and density functional perturbation theory^52^ as implemented in the Quantum Espresso package^53^, respectively. For the exchange correlation functional, the generalized gradient approximation^54^ of PBE^55^ was used with a plane-wave basis-set cutoff of 500 eV, and the Heyd–Scuseria–Ernzershof exchange-correlation functional (HSE06)^56^ and quasi-particle (GW_0_) calculations^57^,^58^ were conducted to estimate more accurate band gaps. BZ integration was performed with a grid sampling of 40 × 40 × 40 *k*-points in DFT and HSE06 calculations. We also conducted DFT and HSE06 calculations by entering a *k*-point list for the portion of the BZ near the band gap, explicitly to achieve a dense grid of 100 × 100 × 100 *k*-points. The Bethe–Salpeter equation^59^ was used to compute the imaginary portion of the dielectric function by including electron-hole interactions, which is implemented in the ABINIT software^60^.

### Data availability

All the relevant data that supports the findings and conclusion of this work are available from the corresponding authors on request.

## Additional information

**How to cite this article:** Zhao, Z. *et al*. Properties of the exotic metastable ST12 germanium allotrope. *Nat. Commun.* **8,** 13909 doi: 10.1038/ncomms13909 (2017).

**Publisher's note**: Springer Nature remains neutral with regard to jurisdictional claims in published maps and institutional affiliations.
